# Supplementary material for: Immune System Stimulation Reduces the Efficiency of Whole-Body Protein Deposition and Alters Muscle Fiber Characteristics in Growing Pigs
Source: Animals (Basel). 2019 Jun 6;9(6):323. doi: 10.3390/ani9060323 (PMC6617207; doi:10.3390/ani9060323)
Supplement: Supplementary file 1 [file animals-09-00323-s001.pdf]

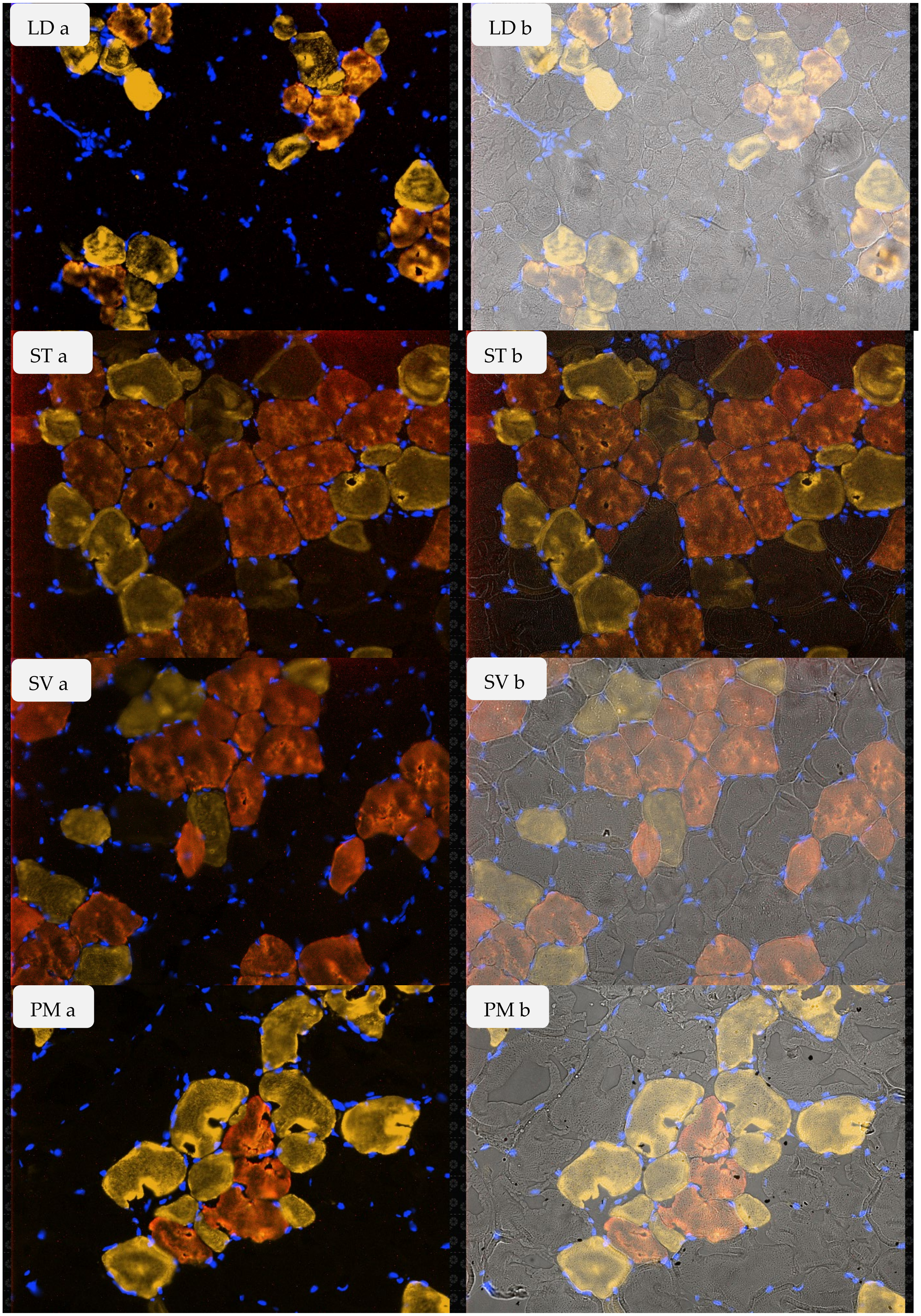
LD a
LD b
ST a
ST b
SV a
SV b
PM a
PM b

Figure S1. Example images of sections of *longissimus dorsi* (LD), *semitendinosus* (ST), *serratus ventralis* (SV) and *psoas major* (PM) with (a) or without (b) transil-lumination. Immunohistochemical staining of cross sections was performed using anti-myosin heavy chain (MHC)-IIB IgM (10F5), anti-MHC-IIA and anti-MHC-I IgG1 (BF-35), and anti-MHC-I IgG2b (BA-D5). Slides were imaged using an inverted fluorescence microscope with a UV light source and a CoolSnap ES2 monochrome camera. Images were artificially colored and analyzed using NIS Elements Imaging software.
